# The Complex Interplay between Chronic Inflammation, the Microbiome, and Cancer: Understanding Disease Progression and What We Can Do to Prevent It

**DOI:** 10.3390/cancers10030083

**Published:** 2018-03-20

**Authors:** Heather Armstrong, Michael Bording-Jorgensen, Stephanie Dijk, Eytan Wine

**Affiliations:** 1 CEGIIR, University of Alberta, Edmonton, AB T6G 2X8, Canada; bordingj@ualberta.ca (M.B.-J.); sispragu@ualberta.ca (S.D.); wine@ualberta.ca (E.W.); 2 Department of Pediatrics, University of Alberta, Edmonton, AB T6G 1C9, Canada; 3 Department of Physiology, University of Alberta, Edmonton, AB T6G 1C9, Canada

**Keywords:** cancer, microbiota, inflammation, diet

## Abstract

Cancer is a multifaceted condition, in which a senescent cell begins dividing in an irregular manner due to various factors such as DNA damage, growth factors and inflammation. Inflammation is not typically discussed as carcinogenic; however, a significant percentage of cancers arise from chronic microbial infections and damage brought on by chronic inflammation. A hallmark cancer-inducing microbe is *Helicobacter pylori* and its causation of peptic ulcers and potentially gastric cancer. This review discusses the recent developments in understanding microbes in health and disease and their potential role in the progression of cancer. To date, microbes can be linked to almost every cancer, including colon, pancreatic, gastric, and even prostate. We discuss the known mechanisms by which these microbes can induce cancer growth and development and how inflammatory cells may contribute to cancer progression. We also discuss new treatments that target the chronic inflammatory conditions and their associated cancers, and the impact microbes have on treatment success. Finally, we examine common dietary misconceptions in relation to microbes and cancer and how to avoid getting caught up in the misinterpretation and over inflation of the results.

## 1. Introduction

**Figure 1:**
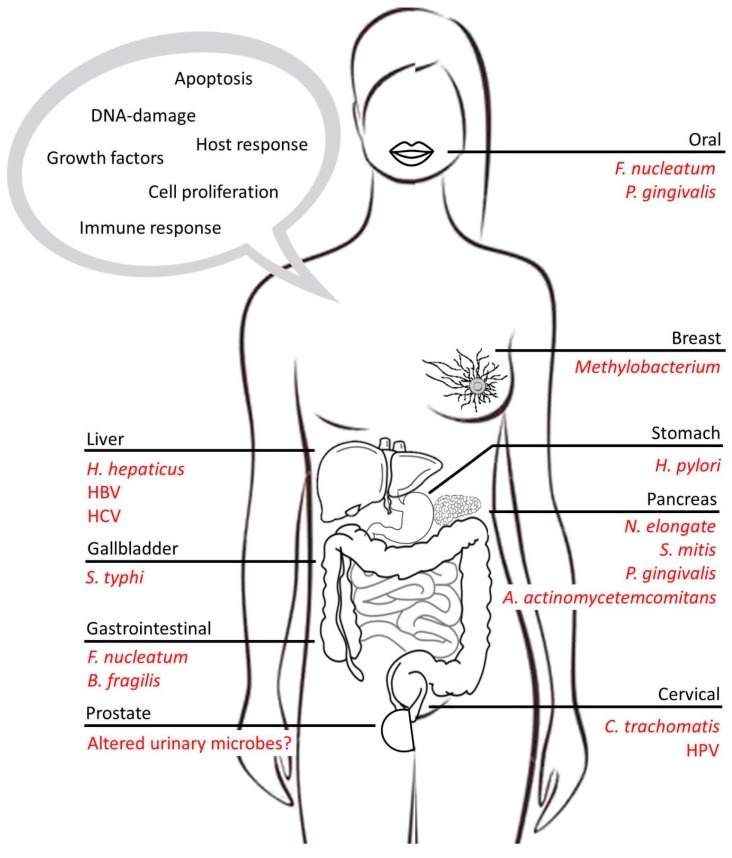
Highlighting the role of microbes in cancer and the key pathways mediated by microbes within host cells.

The link between inflammation and cancer was first proposed by the German physician, Rudolf Virchow, in 1863 upon his discovery of white blood cells (leukocytes) within cancerous tissues [1]. While Virchow hypothesized that cancer could originate at sites of chronic inflammation [1], the last 150 years have seen a dramatic progression in our understanding of this topic (Figure 1: timeline of key dates in the understanding of inflammation and cancer). It was first proposed that a combination of specific irritants and tissue injuries, resulting in inflammation, increases the proliferative capacity of cells in the involved area, leading to sites of carcinogenesis [1]. The hallmark abnormal growth pattern of cells (neoplasia) associated with carcinogenesis is now known to involve far more than increased cell proliferation; Growth factors [2,3], DNA-damage-promoting agents [4,5], activated stroma [6], and a micro-environment rich in inflammatory cells [7] all contribute [1,8]. Advances in technology have enhanced our appreciation of the role of microbiota as environmental factors, beyond just causing infections. Interestingly, chronic inflammation involving microbes precedes development of a tumor site in up to 10–20% of cancers [9,10]. Furthermore, this inflammation can play a role in established tumors and in response to cancer therapeutics [9,10]. Research continues to improve our understanding of the molecular mechanisms, which mediate the complex relationship between inflammation, the microbiome, and cancer. In this review, we will focus on our current understanding of these relationships and what we can do to prevent disease progression. As novel approaches to treat and prevent cancer are critically needed, better defining the roles of microbes and inflammation can offer such unique opportunities in the future.

## 2. Inflammation and Disease

Inflammation represents a host response resulting from a number of factors including, but not limited to, pro-inflammatory mediators, environmental toxins, and chronic infection [11,12,13]. Importantly, in a cancer setting, inflammation plays a role in physiological processes such as controlling infection and wound healing, which are instrumental in disease development and progression, and have been highlighted well in other reviews [13]. Briefly, in response to tissue damage, host cells release various chemical signals, which initiate activation and directed migration of leukocytes (neutrophils, monocytes, and eosinophils) to the site of damage to repair afflicted tissues. The first leukocytes to be recruited in response to chemotactic signaling are neutrophils, which are responsible for stimulating the repair process and initiating inflammation [13,14]. This influx is followed by monocytes which, upon entry into the tissue, differentiate into macrophages. Local endothelial, epithelial, and mesenchymal cells are largely influenced by the growth factors and cytokines produced by activated macrophages. Chemokines, a family of chemotactic cytokines, direct the recruitment of specific leukocyte effector cells, thereby dictating the progression of the inflammatory response [1,15,16,17]. This is particularly important in chronic disease as abnormal, unremitting, inflammatory response can result from dysregulation of any of the cytokine/chemokine signaling factors. In contrast, site-specific inflammation in a normal setting is often thought to be self-limiting, due to the ensuing production of anti-inflammatory cytokines following the influx of pro-inflammatory cytokines. When dysregulated this process results in persistence of initiating factors or failed resolution of the inflammatory response. The subsequent chronic inflammation leads to subversion of cell death and repair programs and ultimately, contributes to cancer pathogenesis.

Studies have shown that use of agents, including non-steroidal anti-inflammatory drugs, is associated with protection against tumor growth and development, suggesting that chronic inflammation predisposes patients to various forms of cancer [1,13,14,18,19]. Further evidence has been provided by studies that show that most neoplastic tissues express an inflammatory component within their tissue microenvironment. This includes cancer types not obviously associated with inflammation and involves characteristic factors such as tissue remodeling, angiogenesis, tissue infiltration of leukocytes, predominance of tumor-associated macrophages (TAMs), and an increased presence of cytokines (tumor necrosis factor [TNF], interleukin [IL]-1, IL-6) and chemokines (CCL2 and CXCL8).

## 3. Microbes as Drivers of Chronic Inflammation: The Link to Cancer

Microorganisms colonize mucosal surfaces at birth and are involved in homeostatic processes including immune development and education and host defense through critical host-microbe, interkingdom signaling [20,21]. This is especially well recognized in the gut, which includes a similar number of microbial cells to the total number of human cells in the body, as evidenced by a profoundly underdeveloped immune system in germ-free animals [22]. Microbes are critical to many chronic inflammatory conditions, including, for example, inflammatory bowel diseases (IBD), arthritis, and primary sclerosing cholangitis, all of which are linked to cancer [23,24,25,26]. Indeed, animal models have supported a direct role for microbes in cancer pathogenesis in these conditions [27,28,29]. Following are examples of specific cancers clearly linked to exposure to microbes (Figure 1).

### 3.1. Gastric Cancers

One of the most common examples of inflammation-associated cancer involves the bacterium *Helicobacter pylori*, which can be found in the stomach of up to two thirds of the world’s population [30]. *H. pylori* can promote host inflammatory responses through the production of virulence factors, including cytotoxin-associated gene (Cag)A, which interacts with and activates host inflammatory protein pathways such as MEK/ERK, NF-κB, and β-catenin [31,32,33,34]. *H. pylori* was the first pathogen to be considered a carcinogen by the World Health Organization and has been identified as the culprit responsible for 70% of gastric adenocarcinomas, along with chronic gastritis, and lymphomas of the mucosa-associated lymphoid tissue (MALT) [35,36]. Interestingly, animal model studies have shown that mice infected with *H. pylori* alone develop more extensive tumor profiles when compared to their germ-free and antibiotic-treated control populations [37,38]. Approximately 3% of patients infected with *H. pylori* will develop gastric cancer, suggesting that *H. pylori* may not be sufficient to cause cancer and is likely not acting alone in the development of inflammation-associated cancer. Infection by Epstein-Barr Virus (EBV) has also been associated with development of gastric cancers through aberrant gene methylation including *RUNX1*, *RBM5*, and *PSME1* [39,40,41]. The outcome of infection also depends on environmental factors (e.g., smoking), host genetic predisposition (polymorphisms in genes encoding IL-1B, IL-10, TNF), and the crosstalk between strain-specific bacterial virulence factors (*cag*PAI, T4SS, CagA) [36,42,43,44,45]. Although gastric cancer incidence has been declining over the last few decades (likely due to an improved understanding of their cause and an increasing number of available therapeutics) they remain the second leading cause of cancer-related deaths worldwide [46].

### 3.2. Liver Cancers

Primary liver cancers, including the most common form known as hepatocellular carcinoma (HCC), remain the third leading cause of cancer-related deaths worldwide [47] and have been associated with hepatic injury and inflammation in up to 90% of cases [48]. Persistent chronic inflammation of the liver has been associated with liver fibrosis, cirrhosis, and subsequent HCC. Further studies have identified infection with Hepatitis B (HBV) or C (HCV) viruses, which result in liver inflammation, leading to increased risk of HCC development by almost 20-fold [47]. Both HBV and HCV are thought to result in liver damage and inflammation through the active response of CD8+ T- and natural killer (NK) cells [49,50]. Furthermore, in response to HBV and HCV viral infection, macrophage and neutrophils produce reactive oxygen species (ROS) and nitrogen compounds as a part of their inflammatory response, leading to DNA-damage associated with HCC and other cancers [49,50,51]. More recent studies have also implicated gut microbes such as *Helicobacter hepaticus* in the development of liver cancers, measured by tumor growth and induction of nuclear factor-κB (NF-κB) in response to intestinal colonization by *H hepaticus* [52]. 

### 3.3. Pancreatic Cancers

The increased risk of pancreatic cancer is also believed to be due to chronic inflammation and hyper-proliferation of pancreatic stellate cells [53]. Chronic pancreatitis can be brought on by a series of factors including environment factors (e.g., smoking), genetic predisposition, metabolic abnormalities, and infection [54]. Evidence accumulating from more recent studies in both human and animal models has suggested that specific microbes are also linked to inflammation associated with pancreatic cancer [55,56]. The presence of the periodontal pathogens *Neisseria elongate*, *Streptococcus mitis*, *Porphyromonas gingivalis*, and *Aggregatibacter actinomycetemcomitans* were closely associated with an increased risk of pancreatic cancer. Pancreatic cancer is characterized by very poor prognoses with a 5-year survival rate below 7%. Identification of specific microbes involved may provide important biomarkers to detect risk groups and aid in the development of targeted treatments towards these periodontal microbes, potentially preventing cancer development in the first place. 

### 3.4. Colorectal Cancer

One of the most prevalent groups of chronic inflammatory diseases, IBD, is an important risk factor for the development of colorectal cancer (CRC), which is the third most common malignancy worldwide [10,57]. IBD-associated intestinal inflammation is characterized by site-specific influx of neutrophils, macrophages, and other immune cells [58,59,60]. Cytokines, free radicals, and proteolytic enzymes produced by these cells result in the hallmark inflammation and ulcerations found in IBD patients. Chronic inflammation associated with IBD can initiate tumourigenesis as the infiltrating immune cells within the intestinal tract create a microenvironment composed of elevated amounts of ROS and reactive nitrogen species (RNS) [61,62]. A build-up of ROS and RNS results in DNA damage and exogenous mutagens in surrounding tissues, promoting the initiation of cancer. The profile of cytokines and growth factors produced in IBD are similarly expressed in CRC and are vital for the growth of CRC tumors [10,57]. These include TNF, IL-1, IL-6, IL-17, IL-22, and IL-23 [63,64,65,66,67,68,69,70,71,72,73,74]. IBD is further characterized by dysbiosis or an altered microbiome that is thought to play a role in both the development of inflammation in IBD and progression to CRC. One bacterium, *Fusobacterium nucleatum*, has been shown, through metagenomics analysis, to be enriched in colorectal carcinoma tissues [75]. The direct role of *F. nucleatum* in the development of colorectal carcinoma is still not completely understood but it is clear that they play a significant role in its progression. 

### 3.5. Breast and Prostate Cancer

Breast and prostate cancer are the leading causes of cancer death for women and men, respectively, and recent evidence suggests a role for inflammation and tissue microbes in disease pathogenesis [76,77,78]. The greatest microbial alteration found in breast tissue is a reduced abundance of *Methylobacterium* in breast cancer patients and has been correlated with tumors of greater invasive potential. Further perturbation of microbes whose products metabolize estrogen, and associated hormones cause circulating estrogen levels to increase, ultimately increasing the risk of breast cancer development. 

In men with prostate cancer, the microenvironment of the prostate frequently contains inflammatory cells; therefore, studies have examined the role of inflammation and prostatic infection in prostate cancer development. It is thought that as development of prostate tumors progresses and oxygen supply is depleted, growth of anaerobic bacteria may become more prevalent [79]. Although identification of microbial involvement has proven complicated in prostate cancer, it has been established that inflammatory changes in the prostate microenvironment along with infection of the prostate are associated with epithelial barrier disruption, promoting prostate cancer development and progression [78]. Evidence suggests it is the altered microbiome of the urinary tract that leads to the potential for infection of the prostate [78,80,81]. 

## 4. Microbes and Inflammation

Interestingly, as demonstrated in the examples above, one common trait shared among many chronic inflammatory diseases contributing to cancer is an altered microbiota, or the involvement of specific microbes (bacteria, viruses, fungi) in both inflammation and disease development [82]. A fine balance must be maintained between the beneficial commensal microorganisms and the pathogens attempting to establish themselves within the host. Gut commensals play a role in modulating host immunity, promoting host defense, synthesizing essential vitamins, and processing indigestible components of the hosts diet; while pathogens are involved in disease development and altering the host microenvironment to aid in establishing infection [83,84,85]. This alteration of the host microenvironment can eventually lead to dysbiosis in chronic conditions, thereby decreasing the benefits of the commensal microbiome. 

Dysbiosis of the microbiome of the intestinal tract was observed in many disorders, and thus it is no surprise that there is a link with gastrointestinal carcinogenesis. Under normal physiological conditions, the gastrointestinal epithelial cells are protected by a mucous layer, which contains components that prevent direct interaction with many microbes as well as antimicrobial peptides. This mucous layer acts as a natural barrier, together with the immune system, to maintain microbes from the external environment at a healthy distance from the underlying host tissue [86]. This mucous lining is not a novel characteristic of the gut alone; it is also found in the lung, urethra, anus, and nose, among other organs [86]. However, many inflammatory conditions lead to a disruption of the mucosal layer allowing microbes to come into direct contact with the underlying tissue and epithelium [87]. 

The role of specific microbes in the development of cancer continues to be established [86,88]. If we recall that the presence of specific periodontal pathogens is associated with increased risk of pancreatic cancer, it is not surprising that a connection has also been established between the oral microbiome, such as *Porphyromonas gingivalis* and *Fusobacterium nucleatum*, and oral squamous cell carcinoma [88,89]. As mentioned previously, a number of microbes have been well established for their role in various cancers including *H. pylori* in MALT lymphomas, *H. pylori* and EBV in gastric cancer [41,86,90], *Bacteroides fragilis* and *Fusobacterium nucleatum* in colon cancer [75,91], Human T lymphotropic virus (HTLV-1) in adult T-cell leukemia (ATL) [92,93], *Chlamydia trachomatis* and HPV in cervical cancer [94,95], EBV in Hodgkin’s lymphoma (HL) [96,97], *Salmonella typhi* in gallbladder cancer [98], *Fusobacterium* and HPV in Head and neck squamous cell carcinomas (HNSCCs) [99,100], and KSHV in Kaposi’s sarcoma (KS) [101]. One such study by Yakoob et al. recently demonstrated that patients with B-cell non-Hodgkin lymphoma were colonized with *H. pylori* that displayed increased expression of the outer membrane protein *HopQ*. *HopQ* is a virulence factor that enables *H. pylori* adherence to gastric epithelial cells, establishing a mechanism for increased infection in these patients [102]. However, how are these microbes involved in the development of their associated cancers?

## 5. Mechanism of Action of Microbes Associated with the Development of Cancer

Cancer pathogenesis involves a number of factors including a micro-environment rich in inflammatory cells [7], growth factors [2,3], DNA-damage-promoting agents [4,5], activated stroma [6], induced cell proliferation, and inhibition of apoptosis, among other factors [1,8]. Specific microbes display unique characteristics in their ability to regulate changes in their host niche which can result in an increased risk of associated cancers. Some of these targets and examples of microbes involved are presented below.

### 5.1. Regulation of Immune Cells

It comes as no surprise that under conditions of chronic inflammation the microenvironment would be rich in inflammatory cells; however, the role of immune cells in cancer growth is quite complex. A number of reviews highlight the complex interplay of immune cells and the tumor environment, which is beyond the focus of this paper [103,104,105]. Recent research has demonstrated another layer of depth, examining the role that microbes play in regulating specific processes of immunity in the context of cancer development. For example, the presence of T-cells in cancerous tissues has been associated with an increased patient survival [106,107]. One bacterium, *F. nucleatum,* possesses a virulence factor that can suppress T-cell activity similar to *H. pylori* in gastric cancer. Mima et al. have recently shown that there is an inverse relationship between the numbers of *F. nucleatum* present in a tissue and the amount of CD3+ T-cells [55,108]. This suggests that this bacterium is suppressing T-cell activity in patients, thereby allowing for cancer development, and possibly impacting response to treatment. In fact, many therapies under development aim to mobilize T-cell populations to seek and destroy tumor cells [109], a common goal of immunotherapies (discussed below in more detail). Another study by Kuhn et al. demonstrated *Bacteroidales* to be important for intestinal membrane integrity by stimulating intraepithelial lymphocyte secretion of IL-6; however, while low levels of IL-6 are tolerable, increased levels of IL-6 promotes tumourigenesis by signaling proliferation, angiogenesis, and invasiveness while inhibiting apoptosis [110,111]. Cytokines such as IL-1β, TNF-α, and IL-6 are also produced by cells infected with viruses including Epstein-Barr Virus (EBV), HBV, HCV, and Kaposi’s sarcoma herpesvirus (KSHV), which induces an inflammatory environment and promotes tumourigenesis [112,113,114]. This highlights the importance of elucidating the healthy balance of microbes within the body, and specifically the gut. Recognizing the role of microbes in regulating these immune cells may provide an alternative to immunotherapies through the use of microbe-altering diets and targeted therapeutics. 

Alternatively, in some cancers such as intestinal cancers, the role of microbes may be less direct. While the immune system of a healthy patient displays a level of tolerance for commensal microbes, immune-impairment, such as found in patients with chronic inflammatory conditions, can lead to inappropriate immune responses towards microbes resulting in intestinal tumor growth [115,116].

### 5.2. Growth Factors

Induced expression of growth hormones is mediated in part by microbes that prompt senescent cells to secrete growth factors, which has been shown to enable tumor growth [117]. One study demonstrated that *E. coli* species that produce the genotoxin colibactin, induce tumor growth by increasing production of hepatocyte growth factor in human and mouse colon cancer models [117]. Further studies in colorectal cancer patients and mouse models indicated that an increase in specific *E. coli* strains expressing the *pks* pathogenicity island produce the genotoxin colibactin [118,119]. Colibactin promotes tumourigenesis through the production of growth factors as a result of alterations in the SUMOylation of p53 [118,119]. Transforming growth factor beta (TGFβ) plays a variable role in the regulation of cell growth, proliferation, differentiation, and apoptosis, depending on the cell type and state. In benign cells it has been shown to inhibit cell cycle however, in cancer cells, TGFβ promotes tumor cells growth and metastasis through the promotion of ERK pathways [120]. A number of infectious agents, including influenza A virus, Group A *Streptococcus*, and *Staphylococcus aureus* utilize TGFβ [121,122,123]. Interestingly, blocking growth factor production using TGFβ inhibitors or Smad3 signaling inhibitors results in reduced secretion of extracellular matrix components involved in bacterial binding and therefore has been demonstrated to reduce Group A *Streptococcus* (fibronectin-binding) abundance [122,123]. Furthermore, by reducing the secretion of extracellular matrix proteins, these inhibitors prevent tumor cell dissemination, proliferation, and metastasis [124,125,126,127,128].

Binding of ligands to one of the most well studied growth factor receptors, epidermal growth factor receptor (EGFr), results in activation of cell signaling pathways, causes mass protein phosphorylation, and leads to cytoskeletal reorganization promoting tumourigenesis [129]. EGFr ligands play a role in regulating gastrin production by *H. pylori*, as inhibiting EGFr has been shown to reduce gastrin expression, thereby reducing risk of tumourigenesis [130]. Interaction of microbes, such as *H. pylori*, with host cells modulates host cell signaling pathways involved in the promotion of cancer, such as mitogen-activated protein kinase (MAPK) pathway, integrin-mediated signaling, and heparin-binding EGF-like growth factor (HB-EGF) pathways [130,131,132]. Interestingly, use of probiotics (microbes with potential beneficial effects) such as *E. coli* Nissle 1917, leads to epithelial wound healing through interactions of the microbes with EGFr, which would suggest potential therapeutic use of this system for gastrointestinal mucosa, and other epithelial tissue repair [133]. This highlights the important interplay between host cells and both the commensal and pathogenic microbes found within the host and the host microenvironment [134]. 

### 5.3. Promoting the Hallmarks of Cancer

Tumourigenesis has been shown to be directly modulated by specific microbes through the production of toxins including genotoxins. Genotoxins are known to result in the production of tumor-promoting metabolites, and further induce DNA damage. The host cell responses to DNA damage are affected by microbe-produced toxins including, *B. fragilis* toxin, cytotoxic necrotizing factor 1, cytolethal distending toxin (CDT), and colibactin, which have been implicated in tumorigenesis [118,135]. CDT is produced by a number of species associated with gastric cancer, colorectal cancer, and gallbladder cancer, such as *E. coli*, *S. typhi*, and *H. pylori* [136]. Genomic instability may also result from metabolites (hydrogen sulfide and superoxide radicals) produced by microbes [137,138]. For example, colorectal tumors develop in mice in response to large amounts of superoxide radicals produced by the bacterium *Enterococcus faecalis*, which have been shown to result in chromosome instability and double-strand DNA breaks [139,140]. A number of microbial species including *E. coli*, *Campylobacter jejuni*, *Aggregatibacter actinomycetemcomitans*, *Haemophilus ducreyi*, *Shigella dysenteriae*, *Helicobacter hepaticus*, and *S. enterica* produce the genotoxin compound of the CDTs family, which display potent DNase activity causing DNA lesions and apoptosis [141]. 

Protein A of *S. aureus* is known to stimulate phosphorylation of EGFr, which in turn leads to phosphorylation and activation of ADAM17 [129,142,143,144]. Stimulation of EGFr results in cell proliferation, increased survival, cellular differentiation, adhesion, and migration [145]. Regulation of EGFr and its associated pathways has been linked to gastric, colon, liver, lung, and pancreatic cancers [146,147,148,149,150,151]. For example, in an inflammatory microenvironment, activation of signal transducer and activator of transcription (STAT)3 by IL-6 promotes proliferation of pre-malignant cells and inhibits apoptosis [148,149,152]. Proliferation is promoted by STAT3 through upregulation of cyclin D1, cyclin D2, and cyclin B cell cycle regulators, along with the MYC transcription factor [148,149,153]. Furthermore, STAT3 upregulates expression of the anti-apoptotic genes BCL2 and BCL2-like 1 (BCL2L1) resulting in reduced apoptosis and promotion of tumourigenesis. 

## 6. Microbes and Inflammation in Cancer: Means of Treatment and Prevention

### 6.1. Response to Chemotherapy

Several currently available cancer therapeutics are listed with summaries provided by the National Cancer Institute (available at: https://www.cancer.gov/about-cancer/treatment/drugs) while those currently undergoing clinical trial for possible FDA approval have been listed by the National Institute of Health (available at: https://clinicaltrials.gov/ct2/results?cond=Cancer&term=&cntry=&state=&city=&dist=). Platinum-based antineoplastic compounds, often referred to as platins, are used to treat up to 50% of all cancer patients [154,155,156]. Studies performed in animal models demonstrate that the antitumor activity of platins is reduced in the absence of commensal microbes and although the compounds remain detectable within tumors, DNA damage is dramatically attenuated [157]. These agents function to induce apoptosis and cytotoxicity in tumor cells by inhibiting DNA replication through the formation of double-stranded breaks and intrastrand platinum-DNA adducts [158,159,160,161]. This process is driven by microbes that promote production of ROS via NADPH oxidase 2 (NOX2) by tumor-infiltrating myeloid cells [157]. For example, addition of *Lactobacillus acidophilus* to germ-free mice restores the antitumor activity of cisplatin [162]. Interestingly, platins also result in severe intestinal toxicity, nephrotoxicity, and peripheral neuropathy, which can be attenuated by the addition of the probiotic *L. acidophilus* [162,163]. It is not surprising, based on these data, that targeting the microbiota through the use of prebiotics (complex carbohydrates that promote growth of beneficial microbes), probiotics, and symbiotics (combined probiotics and prebiotics) has the potential to limit toxicity while improving therapeutic efficacy [164]. 

Microbes play an important role in regulating the pharmacokinetics, toxicity, and mechanism of action of chemotherapeutics [134,165]. For example, gut microbes metabolize injected compounds, such as the intravenous topoisomerase I inhibitor, CPT-11 (tissue carboxylesterase transforms Irinotecan), used for colorectal cancer treatment [166]. CPT-11 is converted to its active form, SN-38, and further detoxified to inactive SN-38-G in the liver by UDP-glucuronosyltransferases [166]. In the gut, SN-38-G is converted back to SN-38 by bacterial β-Glucuronidase, most often associated with Firmicutes, particularly *clostridia*, resulting in intestinal toxicity, diarrhea, and intestinal inflammation [166,167,168,169]. These symptoms can be prevented in patients with high abundance of β-glucuronidase-positive bacteria through the use of targeted antibiotics or bacterial β-glucuronidase-specific inhibitors [169]. Further, bacterial enzyme production is altered in response to xenobiotics, including chemotherapeutics, and these enzymes regulate bioavailability of many oral compounds [170,171]. Another example relates to gemcitabine, a chemotherapeutic agent commonly used for treating pancreatic ductal adenocarcinoma. Gammaproteobacteria were found to have the capacity to metabolize gemcitabine into an inactive metabolite through the bacterial enzyme cytidine deaminase (CDD_L_); this effect was reversed by use of antibiotics [172]. Interestingly, viruses including EBV have developed techniques to augment viral expansion by encoding the Epstein-Barr virus (EBV) homologue of the BCL-2 proto-oncogene, BHRF1, which prevents apoptosis of the infected cells and confers a strong level of chemoresistance [173,174,175]. As small molecule inhibitors of Bcl-2 do not target BHRF1, studies have examined targeted inhibitors of BHRF1, such as BINDI, and have demonstrated induced apoptosis in EBV-infected cancer cell lines and reduced tumor growth in xenograft models of EBV-positive human lymphoma [175,176]. These studies suggest viral-targeted therapies may improve the prognosis of viral-associated cancers.

Microbial composition is also altered in response to some antitumor agents, such as the alkylating agent cyclophosphamide (CTX) [177,178]. CTX allows gut microbes, including *Lactobacillus johnsonii*, *Lactobacillus murinus*, and *Enterococcus hirae* to translocate across the epithelial barrier by increasing mucosal permeability in the gut [177,178]. Treatment with CTX also results in reduction of *Treponema* (Spirochaetes), *Clostridium* (Firmicutes), *Roseburia* (Firmicutes), *Coprococcus* (Firmicutes), and *Lachnospiraceae* (Firmicutes), and increases abundance of *L. johnsonii*, *L. murinus*, *E. hirae*, and *L. reuteri* [177]. This shift in microbes results in activation of memory T helper (Th)1 and pathogenic pTh17 cells and CTX antitumor effects can be reduced in mice treated with targeted antibiotics [177,179]. 

### 6.2. Immunotherapy

One of the most successful interventions for patients suffering from metastatic disease following treatment failure is immunotherapy, which recent studies have demonstrated to be modulated by the composition of the gut microbiota [157,180,181,182]. Immune checkpoint inhibitors, such as the programmed cell death protein 1(PD-1) inhibitors, are highly effective in some patients with advanced melanoma, non-small cell lung cancer, and renal cell carcinoma by suppressing the interaction of T inhibitory receptors with their associated ligands on tumor cells, thus promoting cytotoxic and memory T lymphocyte-mediated immune responses [183,184]. Antibiotic treatment in patients prior to anti-PD-1 therapy reduced progression-free and overall survival of patients compared to those who had not taken antibiotics. Interestingly, increased abundance of the gut bacterium *Akkermansia muciniphila* was associated with improved clinical response to anti-PD-1 treatment, likely due to T-cell mediated response promoted by release of IL-12 in response to *A. muciniphila* [183]. Increased abundance of the Clostridiales order and the *Faecalibacterium* genus, associated with increased CD8+ T cells, demonstrated greater response to anti-PD-1 therapy and experienced longer progression-free survival [184]. Members of the Bacteroidales order were associated with poorer outcomes and demonstrated diminished cytokine response and increased levels of myeloid-derived suppressor cells and circulating regulatory T cells associated with reduced antitumor immunity [184]. Other studies suggest that *Bifidobacterium* may reduce tumor growth through manipulation of dendritic cell function, improving the anti-tumor activity of cytotoxic T cells [181]. In many ways, bacteria that would typically worsen immune-mediated conditions have a favorable effect in the setting of cancer immunotherapy and vice versa. The reason for this is likely the need for a proinflammatory response against the tumor to promote effectiveness of immunotherapy [185]. The efficacy of an anti–cytotoxic T-lymphocyte-associated protein 4 (CTLA4) immunotherapeutics can also be improved by enhancing the antitumor activity of cytotoxic T-cell populations with increased abundance of *Bacteroides thetaiotamicron* and nontoxigenic *Bacteroides fragilis* [182]. The polysaccharide A (PSA) of *B. fragilis* has been shown to regulate the balance between effector and regulatory T cells, resulting in enhance antitumor immunity and promoting an anti-inflammatory state [182,186,187]. Interestingly, the *B. fragilis* toxin (BFT) has recently been shown to trigger pro-carcinogenic inflammatory pathways in colonic epithelial cells through IL-17R, NF-κB, and STAT3 indicating the potential pathogenic role this bacterium may play [188].

### 6.3. Diet as a Bridge between Microbes and Cancer

**Figure 2:**
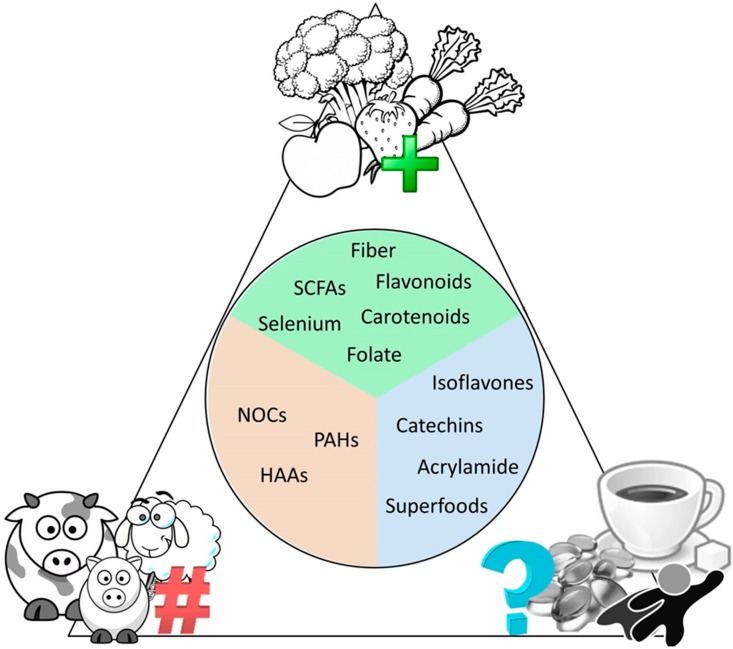
The current consensus on dietary interventions in cancer. What the research studies performed on dietary factors say about their positive, negative, or unknown effects on cancer risk.

While there are innumerous cancer therapeutic interventions available to date, one of the key intervention strategies remaining to be fully elucidated is the role of diet both in disease development and prevention, as well as the role of diet in regulating the human microbiome (Figure 2). It has been well established that a diet rich in fruit and vegetables reduce the risk of lung, throat, larynx, and mouth cancers; unfortunately, diet can be very difficult to measure and control in an experimental setting in a human cohort due to differences in portion size, food availability, and adherence to the particular diet [189,190,191,192]. Fruits and vegetables contain a large number of nutrients including folate, vitamin C, vitamin E, carotenoids, flavonoids, selenium, and natural fibers—all associated with reduced risk of gastrointestinal cancers [193,194,195]. An important series of studies has demonstrated that the key nutrients found in fruits and vegetables do not reduce cancer risk when taken as supplements and may in fact display harmful effects in these instances [196,197]. The adaptation of microbiota to dietary intake has been seen in a Japanese diet rich in seaweed, which has been associated with promotion of the *Bacteroides plebeius* in the human gut to acquire enzymes for seaweed digestion from the marine bacteria *Zobellia galactanivorans* [198]. Recent studies in human breast cancer cell lines have demonstrated that the polysaccharides (SWP1 and SWP2) isolated from the brown seaweed *Sargassum wightii* (SWP) significantly reduced cell proliferation and induced apoptosis in a dose-dependent manner [199]. 

As the gut microbiota clearly plays an important role in inflammation and cancer prevention, introduction of a diverse number of beneficial live bacteria and yeast through probiotics are a promising option for use in establishing and promoting a healthy microbiota. Probiotic bacteria have been shown to display anti-cancer properties in a number of studies by suppressing the growth of microbes involved in the production of mutagens and carcinogens, altering metabolism of carcinogens, protecting DNA from oxidative damage, and regulating the immune system [200]. Natural fermented milk products such as Kefir have been shown to inhibit breast cancer cell proliferation in vitro when compared to normal breast epithelia [201]. Specific studies have elucidated the molecules produced by probiotic microbes which are responsible for their anti-tumor effects. One such study by Konishi et al. identified ferrichrome as the molecule produced by *Lactobacillus casei* which is responsible for the probiotic microbes tumor-suppressive effects [202]. The anti-tumor effects of ferrichrome in colon cancer cells were shown to be even greater than cisplatin and 5-fluorouracil and demonstrated less effect on non-cancerous cells in vitro when compared to either of these clinical agents [202]. While these, and other, results are promising, there are over 400 probiotic strains within the human intestine and there currently remains a lack of clear guidelines on appropriate timing for use of probiotics along with a lack of clear evidence to determine which probiotics are most effective. The effectiveness of a probiotic supplement is dependent on species diversity, dose, and disease status of the patient [203]. To date, clinical studies have shown that probiotics are effective in patients with a number of ailments including acute infectious diarrhea, antibiotic-associated diarrhea, *Clostridium difficile*-associated diarrhea, hepatic encephalopathy, ulcerative colitis, irritable bowel syndrome, functional gastrointestinal disorders, and necrotizing enterocolitis [203]. Probiotics have not been shown to be effective in Crohn disease or acute pancreatitis [203]. While in vitro and in vivo studies remain promising, a great deal of clinical research remains to be done to improve our understanding of the effectiveness of probiotics. Lescheid et al.

A number of studies have also associated a diet high in red meat, that when consumed more than twice a week may increase the risk of gastrointestinal, pancreatic, and prostate cancers [204,205,206,207,208,209,210]. In these studies, red meat includes fresh, minced, and frozen lamb, beef, pork, and processed meats including, sausages, bacon, ham, and salami [204]. Research recommends that a maximum of 70 g or 0.15 lb of red meat should be consumed daily to reduce this risk. While a causative link remains to be identified, some research suggested that the haem pigment, found only in red meat, could stimulate production of cancer causing N-nitroso compounds (NOCs) by gut bacteria; the nitrites and nitrates used as preservatives in processed meats can also be converted to NOCs within the body. Alternatively, heterocyclic aromatic amines (HAAs) and polycyclic aromatic hydrocarbons (PAHs) formed when cooking meat at high temperatures, may result in increased risk of cancer [207,211,212].

One key nutrient group under continued investigation is the bacterial metabolites known as short chain fatty acids (SCFAs). In the human gut, dietary fibers are fermented by microbes, resulting in the formation of predominantly acetate, propionate, and butyrate SCFAs [213,214]. SCFAs, which generally have protective roles, enter into the colonic epithelium at varying rates through diffusion and carrier-mediated mechanisms; however, in patients with cancer or chronic inflammatory diseases, such as colitis and arthritis, altered expression of these transport proteins results in reduced uptake of SCFAs [215]. Interestingly, supplementation with acetate has been shown to reduce inflammation in vivo peripherally in arthritis, and acetate stimulation in vitro reduced pro-inflammatory surface receptors on human neutrophils [216]. Furthermore, increasing the fermentable fiber inulin in the diet has been shown to significantly elevate concentrations of SCFA and lower the ratio of omega 6 to 3 essential fatty acids (EFAs) in mice, which has previously been associated with reduced incidence of cancer, inflammation, and other chronic diseases in humans [217]. Mouse model studies demonstrate that inulin-type fructan dietary supplementation increases propionate levels in the portal vein and slows infiltration of transplanted cancer cells [218]. In vitro exposure of these cancer cells to propionate slowed proliferation compared to controls [218]. In vitro treatment of human colon cancer cells with the SCFA butyrate increased expression of the cyclin dependent kinase inhibitor, p21 [219], which has previously been shown by immunohistochemistry to be lost in 79% of colon cancer tumors [220]. As SCFAs are obtained by dietary fibers, these findings provide insight into the results of the Aune et al. metaanalysis that described increased fiber intake is associated with a reduced risk of colorectal cancer [221].

Other micronutrients of note in cancer prevention include isothiocyanates and lignans. The isothiocyanate glucoraphanin in cruciferous vegetables is antineoplastic and anti-inflammatory after activation to the bioactive sulforaphane by a heat sensitive enzyme [222,223]. If the converting enzyme, also present in cruciferous vegetables, is heat destroyed in cooking, conversion to sulforaphane relies on metabolism by the gut microbiome [223]. There is great variability between individuals, with the ability to convert sulforaphane into the active form found to range from 1% to 40% [223]. Sulforaphane has been demonstrated to inhibit malignant growth in various cancer types and has recently been shown to inhibit proliferation of gefitinab resistant lung cancer cells suggesting combination of sulforaphane and gefitinib may provide a more effective therapy for lung cancer [224]. It is very important to note that as with all dietary factors, a large proportion of sulforaphane (up to 70%) is excreted in the patients urine within the first 24 h helping explain its ability to prevent bladder cancer invasion [225,226]. Furthermore, while doses ranging from 1–12 μM have been shown to display antitumourigenic effects in cell lines, oral doses of 150 μM or greater are required to elicit response [224,225]. Beverage formulation of sulforaphane was prepared by re-hydrating previously lyophilized 3-day-old broccoli sprout powders produced from specially selected BroccoSprouts seeds (DM1999B) boiled and aqueous extracted [225]. Lignans, found in plant parts such as seed coats and bran are associated with decreased breast and colon cancer, and are converted into the anti-tumor enterolignans, through metabolism in separate steps by several unique gut bacteria [227]. Enterolignans are associated with a decreased risk of colorectal cancer among women in the EPIC study, but a slight increased risk of prostate cancer in men [228]. Recent studies in laboratory models of colitis in mice have shown that brown rice, a great source of insoluble fiber, is able to alleviate inflammatory symptoms therefore, offering potential to reduce risk of inflammation-associated colorectal cancers [229]. 

A Dietary Inflammatory Index (DII^®^) was developed to categorize patient diets according to inflammatory potential, in which a higher score equates to a greater risk of inflammation [230]. A number of studies support the importance of establishing an anti-inflammatory diet to prevent the development of cancers, especially those of the gastrointestinal tract [230]. Many foods listed in the DII^®^ have been shown to display anti-inflammatory properties, including certain flavonoids (found in many fruits and vegetables) and the spices and herbs: saffron, turmeric, oregano, and eugenol (from cloves) [230,231,232]. Pro-inflammatory foods in the DII^®^ include dietary cholesterol, trans fat, and saturated fat [230,231,232]. Studies performed in rats on a high sucrose diet demonstrate that diet supplementation with the flavonoids quercetin and resveratrol reduces the ratio of Firmicutes/Bacteroidetes and ultimately reduces weight gain and insulin resistance compared to rats on a high sucrose diet without supplementation [233]. Microbiota changes are also found in response to supplementation with the active compound of turmeric, curcumin, including reduction in Prevotellacea and increased Bacteroidaceae and Rikenellaceae [234]. Other studies evaluating curcumin supplementation in the IL-10^−/−^ mouse model of colitis associated cancer demonstrated increases in *Lactobacillus*, which has previously been shown to help prevent colorectal cancer [235,236]. 

### 6.4. Common Dietary Misconceptions

While several fantastic studies have been completed linking diet to microbes, inflammation, and their associated cancers, it is important to distinguish these from several popular theories, often referenced in discussion with patients and researchers alike. Many of these dietary factors have little to no scientific evidence to support their role in disease promotion or prevention and are an over-extrapolation of very specific scientific findings. For example, as mentioned earlier, vitamin supplements do not necessarily provide the same health benefits as naturally-obtained vitamin sources and several clinical trials examining the effect of vitamin supplements in cancer have found that high doses of certain supplements can in fact increase cancer risk [196,197,237]. The Cochrane Collaboration, which continues to examine results from a series of clinical trials, has found that results from over 80 clinical trials prove that vitamin supplements pose either a neutral effect or increased health risk with no health benefit compared to a diet without supplements [238,239,240,241]. It is important to remember that the microbes in our gut participate in the production and bioavailability of many dietary nutrients, including SCFAs [213,214,242]; therefore, maintaining a healthy, balanced flora is essential for maintaining balanced nutrient levels. These studies demonstrate that supplements do not substitute a healthy balanced diet high in fruits and vegetables, although for some patients, supplements are essential to combat a lack of nutrient. Examples of this include calcium supplementation in patients taking steroids [243]; folic acid supplementation for women planning to have a baby [244]; vitamin D supplementation in those at risk of low vitamin D levels including children under 5, adults over 65, pregnant or breastfeeding women, or those with sun sensitivities, among others [245]. 

Further examples include the chemical acrylamide and artificial sweeteners as cancer causing agents, and calcium as an anti-tumourigenic agent. While evidence from animal models suggests a link between these examples and cancer risk [246,247,248,249,250], evidence from human studies is weak [251,252,253,254] and requires more diligence in study design to understand any potential link. Some evidence has demonstrated that microbes, namely bacteria, produce the enzyme, amidase, which decomposes acrylamide [255]. Furthermore, acrylamide appears to be utilized as a carbon source for lactic acid bacteria [255,256]. Artificial sweeteners are commonly used in a variety of foods and drinks and the most extensively studied sweeteners including saccharin and aspartame have been shown to alter microbial composition [248,257,258,259,260]. Calcium is important for tooth and bone health and has further been shown to play a role in cell structure, motility, and transport in a variety of microorganisms [261]. While there is little direct evidence of the role these agents play in direct regulation of microbes in inflammation and cancer, research continues to uncover more evidence to elucidate the mechanisms of their interactions.

An interesting new term in recent years coined “superfood” has been used to describe foods with apparent health benefits and is often applied to foods including blueberries, broccoli, raspberries, and green tea, among other foods. While the sales pitch of this term has suggested these foods can prevent diseases, including cancer, it is simply a marketing tool with very little scientific basis to back these claims; marketing distorts scientific findings, over generalizing and selectively ignoring negating data. While we highlighted earlier the importance of a well-balanced diet to reduce risk of cancer, it is incredibly unlikely that a single food item or superfood will have any major effect alone. These foods are often labeled superfoods as they contain natural chemicals, which have been shown to display positive health effects in a laboratory setting such as antioxidants, vitamins, and minerals, some of which were listed earlier [262,263]. It is important here to recall however that the amount of many chemicals found in foods are trace amounts compared to their purified and isolated forms used in research. These isolated chemicals often behave very differently in a test tube used in a laboratory setting in animals or cell culture methods and often these experiments require very high doses of purified chemical to demonstrate any effect. Extensive studies have been performed on green tea as it contains high levels of polyphenols, including catechins, and anti-inflammatory flavonoids, which have been demonstrated to be beneficial in inhibiting cancer growth in a laboratory setting [264,265,266]. Furthermore, while both black and green tea have been demonstrated to inhibit *H. pylori* with no inhibition on the beneficial gut bacterium *L. acidophilus* [267], large scale studies in humans demonstrate no conclusive beneficial association or reduced risk of pancreatic, lung, breast, prostate, stomach, bowel, thyroid, liver, endometrial, laryngeal, bladder, ovarian, kidney, or esophageal cancers [268,269]. One issue with the transition of clinical efficacy from animal models to human trials may be due to limited bioavailability of polyphenols, however more remains to be examined to better identify appropriate dosing and bioavailability for clinical studies [270] Interestingly, studies promoting the benefit of green tea in cancer have shown that microbes including *Pseudomonas* sp., *E. coli*, *Bifidobacterium* sp., *Lactobacillus* sp., *Bacteroides* sp. and, *Eubacterium* sp., utilize polyphenols such as catechins as a carbon source [271,272,273]. Catechins are catalyzed to protocatechuic acid which has been shown to inhibit metastasis in vitro and is naturally found in fruits and vegetables [272,273]. Flavonoids have also been shown to display anti-tumourigenic effects in cells lines and are hydroxylated by microbes including Aspergillus, an abundant fungi of the gut [271,274,275]. The isoflavones found in soy or soya products such as tofu, are plant-based estrogens extracted from soybeans and display similar structure to, but more mild effects compared to human estrogen [276,277,278]. Laboratory studies have demonstrated the potential use of isoflavones to reduce risk of certain hormone-associated cancers, such as prostate cancer, although studies in humans have produced unclear results [279]. More research is needed to identify the link between isoflavones and cancer at this time; however, initial studies suggest, again, a role for intestinal bacteria in the metabolism of soy isoflavonoids [280], demonstrating the importance of identifying the healthy balance of microbes within the human gut in order to better understand how dietary factors can play a role in promoting or preventing cancer. 

## 7. The Take Home Message

Cancer remains one of the most prevalent group of diseases in the developed world and is a leading cause of deaths worldwide. While a great deal has been discovered about carcinogenesis and the role that DNA damage, growth factors, and inflammation play in both cancer development and progression, we have only made it through the tip of the iceberg in our understanding of the role of microbes in maintaining health and altering the host environment for the promotion of inflammation and cancer. Here we have highlighted the prominent studies that have progressed our understanding of the role microbes play, far beyond the gastrointestinal system. These studies demonstrate a role for microbes in almost every cancer including colon, pancreatic, gastric, and even prostate. It remains imperative that we identify the microbes involved in maintaining health, so we may promote their growth and prevent the growth of those identified to be involved in disease development and treatment failure. As technologies (especially high-throughput sequencing) continue to evolve, we expect that data associating microbes with cancer will continue to amount and will stimulate further research and clinical trials.

How can our understanding of microbes, inflammation, and cancer lead to improved outcomes for patients? Promoting colonization of healthy microbes with probiotics, prebiotics, and symbiotics offers promising results for preventing the development of inflammation, along with inhibiting cancer progression and amplifying the effects of specific anti-cancer therapeutics; however, these products will rarely lead to a long-term effect and a more holistic approach may be required. Some beneficial microbes can be introduced by consuming fermented foods including yogurt products or kefirs, fermented kimchi or sauerkraut, or clinically available probiotic products, to name a few. These microbes require the correct nutrients to survive and thrive so simply consuming them may not aid in the quest for ultimate health. As mentioned earlier, a diet rich in fruits and vegetables, and natural fibers is essential for the sustained health and growth of the microbiota. The loss of these microbes, as seen in dysbiosis, can have a large impact on host health. One of these impacts discussed in this review is the progression of cancer.

Together with the emergence of high-tech treatments to manipulate signaling and immune response, such as immunotherapy, as novel approaches to combat cancer, more attention should be paid to “low-tech”, life style changes that can directly impact the long-term risk of cancer through defined metabolic pathways involving microbes. One of the biggest challenges with this area of research is the need for long term studies (over decades) as well as the poor ability to measure diet over time. Metabolomics, which allows precise measurement of small molecules (many of which are derived from microbes, diet, and the interaction between them), could add to the science of this field. Through combining basic, translational, and clinical research (including epidemiologic studies), novel microbe-altering approaches could revolutionize cancer care and prevention by reducing chronic inflammation and progression to cancer.

## Abbreviations

BFT	*B. fragilis* toxin
Cag	cytotoxin-associated gene
CDDL	cytidine deaminase
CDT	cytolethal distending toxin
CRC	colorectal cancer
CPT-11	tissue carboxylesterase transforms Irinotecan
CTLA4	anti–cytotoxic T-lymphocyte-associated protein 4
CTX	cyclophosphamide
DII^®^	epidermal growth factor receptor
EFAs	essential fatty acids
HAAs	heterocyclic aromatic amines
HB-EGF	heparin-binding EGF-like growth factor
HBV	Hepatitis B
HCC	hepatocellular carcinoma
HCV	Hepatitis C
IBD	inflammatory bowel diseases
IL	interleukin
MALT	mucosa-associated lymphoid tissue
MAPK	mitogen-activated protein kinase
NF-κB	nuclear factor-κB
NK	natural killer
NOCs	N-nitroso compounds
NOX2	NADPH oxidase 2
PAHs	polycyclic aromatic hydrocarbons
PD-1	programmed cell death protein 1
PSA	polysaccharide A
ROS	reactive oxygen species
RNS	reactive nitrogen species
SCFAs	short chain fatty acids
STAT3	signal transducer and activator of transcription 3
TGFβ	Transforming growth factor beta
Th	T helper
TAMs	tumor-associated macrophages
TNF	tumor necrosis factor
